# Single-fraction Gamma Knife radiosurgery for pineal region meningiomas: a retrospective single-center cohort of 44 patients

**DOI:** 10.1007/s11060-026-05566-8

**Published:** 2026-04-14

**Authors:** Ali Haluk Duzkalir, Dogu Cihan Yildirim, Mehmet Orbay Askeroglu, Selcuk Peker

**Affiliations:** 1 Department of Neurosurgery, Koc University Hospital, Istanbul, Türkiye; 2 Koc University Hospital, Gamma Knife Center, Istanbul, Türkiye; 3 Department of Neurosurgery, Koc University School of Medicine, Istanbul, Türkiye

**Keywords:** Adverse radiation effects, Falcotentorial meningioma, Gamma knife radiosurgery, Local control, Pineal region meningioma, Stereotactic radiosurgery

## Abstract

**Purpose:**

To evaluate the long-term efficacy and safety of single-fraction Gamma Knife radiosurgery (GKRS) for pineal region meningiomas (PMs) and to clarify its role as both a primary and adjuvant treatment strategy for this anatomically challenging tumor location.

**Methods:**

This single-center retrospective cohort study included 44 consecutive adults with PMs treated with single-fraction GKRS. Diagnosis was established histopathologically after prior surgery or by predefined MRI criteria with consensus neuroradiological review. Tumor response was assessed volumetrically on serial contrast-enhanced MRI, with regression, stability, and progression defined as ≥ 20% decrease, < 20% change, and ≥ 20% increase in tumor volume, respectively. Local control (LC), progression-free survival (PFS), overall survival (OS), adverse radiation effects (AREs), and Karnofsky Performance Scale (KPS) outcomes were analyzed.

**Results:**

Median clinical and radiological follow-up were 90 and 88 months, respectively. At last radiological follow-up, LC was achieved in 42 of 44 patients (95.5%), including regression in 13 (29.5%) and stability in 29 (65.9%). Kaplan-Meier LC rates were 100% at 2 years, 97.1% at 5 years, and 86.3% at 10 years. PFS rates were 100%, 94.1%, and 72.6% at 2, 5, and 10 years, respectively. AREs occurred in 3 patients (6.8%), all without permanent sequelae. KPS remained stable or improved in all patients.

**Conclusion:**

Single-fraction GKRS provides durable tumor control with low toxicity and preserved neurological function in PMs, supporting its use as a primary treatment strategy in selected patients. Preliminary adjuvant results are encouraging but require validation in larger cohorts.

## Introduction

Meningiomas of the pineal region are rare intracranial tumors arising in the deep posterior midline, where the falx cerebri, tentorium cerebelli, and roof of the third ventricle converge around the pineal gland and quadrigeminal cistern [1–3]. Although they may originate from either the falcotentorial junction or the velum interpositum, these entities have been grouped together in most studies [4]. These tumors are exceedingly rare, accounting for approximately 0.3% to 1.1% of all intracranial meningiomas [5]. Despite their usually benign histology, their deep location and intimate relationship to the deep venous system, brainstem, and cerebral aqueduct make them clinically challenging [6].

Historically, microsurgical resection has been the standard primary treatment for symptomatic lesions in this area [5, 7]. However, resection is technically demanding and may preclude gross total resection (GTR) when the tumor is adherent to or occludes critical deep veins, particularly the vein of Galen or straight sinus [5]. Injury or sacrifice of these structures can result in venous infarction, severe edema, and substantial morbidity [8]. Accordingly, subtotal resection (STR) followed by adjuvant therapy is often accepted as the safer strategy [9].

Given these limitations of aggressive microsurgery, Gamma Knife Radiosurgery (GKRS) has emerged as an important minimally invasive primary or adjuvant treatment option [9]. While GKRS is well established for many intracranial meningiomas, dedicated long-term data for pineal region and falcotentorial lesions remain limited, with most reports restricted to case reports or small series [10, 11].

To address this gap, we evaluated 44 patients with pineal region meningiomas (PMs) treated with single-fraction GKRS in a retrospective single-center cohort. To our knowledge, this represents the largest single-center series and one of the largest published dedicated GKRS cohorts with long-term follow-up for this location. We aimed to characterize long-term tumor control, safety outcomes, and exploratory dose-volume associations in this surgically challenging tumor subgroup.

## Materials and methods

### Study design and patient selection

This single-center retrospective cohort study evaluated consecutive patients with PMs treated with single-fraction GKRS. Patients were eligible if aged ≥ 18 years, had a diagnosis established by histopathological confirmation following prior surgical resection, or by characteristic magnetic resonance imaging (MRI) features in the absence of surgery. Radiological diagnosis required a well-circumscribed, homogeneously contrast-enhancing lesion with dural attachment at the falcotentorial junction or velum interpositum, a dural tail sign, absence of restricted diffusion, and imaging features considered inconsistent with pineal parenchymal tumors, germ cell tumors, solitary fibrous tumor, or metastasis on consensus review by the treating neurosurgeon and neuroradiologist, and had a minimum of 12 months of clinical or radiological follow-up after treatment. Exclusion criteria were multi-session GKRS (≥ 2 fractions), non-meningioma pineal region pathology, prior conventional radiotherapy to the same volume, insufficient imaging for volumetric or dosimetric analysis, or confirmed or suspected Neurofibromatosis Type 2. Falcotentorial junction and velum interpositum meningiomas were not subclassified, as reliable differentiation between these subtypes was not feasible on preoperative MRI; all pineal region meningiomas were analyzed as a single cohort, consistent with prior literature [4].

### Gamma knife radiosurgery technique

All patients were treated using the Gamma Knife platform (Elekta AB, Stockholm, Sweden) with Leksell GammaPlan^®^ software. Tumor volumes were delineated on contrast-enhanced T1-weighted MRI at treatment planning. Prescription dose was individualized primarily according to tumor volume and proximity to the brainstem. Patients with larger tumors or close brainstem proximity received lower marginal doses. The principal dosimetric constraints were a maximum brainstem point dose of ≤ 15 Gy and a brainstem volume receiving ≥ 10 Gy (V10) not exceeding 0.5 cc [12]. No specific dose constraints were applied to the deep venous structures; venous involvement was recorded as an anatomical variable to explore potential associations with treatment response and adverse radiation effects but did not influence dose selection or target delineation. The same dose selection principles were applied to both primary and adjuvant cases. The biologically effective dose was calculated as BED₃ = D × (1 + D/3), assuming an α/β ratio of 3 Gy. Irradiated brain volumes receiving ≥ 10 Gy and ≥ 12 Gy were recorded as V10 and V12, respectively.

### Outcome definitions

Local tumor control (LC) was defined as the absence of radiological progression at last follow-up MRI. Tumor volume was assessed on contrast-enhanced T1-weighted follow-up MRI using semi-automated segmentation with manual correction in Leksell GammaPlan^®^ by the treating neurosurgeon, with consensus neuroradiology review in discrepant cases. Follow-up imaging was obtained on 1.5T or 3T MRI using gadolinium-enhanced T1-weighted sequences, although scanner type was not uniform across the study period. Intra- and interobserver reliability were not formally assessed. Tumor regression, stability, and progression were defined as volumetric changes of ≥ 20% decrease, < 20% change, and ≥ 20% increase relative to baseline, respectively [13, 14]. Overall survival (OS) was measured from GKRS to death from any cause, with censoring at last clinical follow-up. Progression-free survival (PFS) was measured from GKRS to tumor progression or death from any cause, with censoring at last radiological follow-up. Adverse radiation effect (ARE) was defined as new peritumoral edema on post-treatment MRI spatially concordant with the irradiated volume and temporally consistent with expected radiation-induced changes, after exclusion of alternative causes including tumor progression, venous outflow obstruction, and surgery-related changes. AREs were classified as asymptomatic or symptomatic. Advanced imaging (perfusion-weighted MRI, MR spectroscopy) was not routinely used for ARE characterization. Functional status was assessed using the Karnofsky Performance Scale (KPS) at baseline and last follow-up.

### Statistical analysis

All analyses were performed in R (version 4.3.2). Continuous variables are presented as mean ± SD or median (IQR) according to normality assessed by the Shapiro-Wilk test, and categorical variables as frequency and percentage. LC, PFS, and OS were estimated using the Kaplan-Meier method with 95% confidence intervals (complementary log-log transformation). Because death before progression precludes subsequent progression, the cumulative incidence of tumor progression was also estimated using the Aalen-Johansen method with death as a competing event. Both Kaplan-Meier and competing-risk estimates are reported to facilitate comparison with prior literature.

Paired KPS change was analyzed with the Wilcoxon signed-rank test. Spearman’s ρ was used for dose-volume correlations. Statistical significance was set at *p* < 0.05 (two-tailed). The Ki-67 proliferation index, available only in the surgically treated subgroup (*n* = 9), is reported descriptively and was not included in inferential analyses. Given the low number of outcome events (2 progressions, 3 AREs), multivariable regression modeling was not performed, as any such analysis would be unreliable and prone to overfitting. Dose–volume associations are therefore presented as exploratory bivariate correlations only and should not be interpreted as predictive models.

BED₃ is reported descriptively in Table 1 to facilitate comparison with multi-fraction series and was not used as an independent variable in inferential analyses, given its mathematical collinearity with marginal dose in this uniformly single-fraction cohort.

## Results

### Patient characteristics and treatment parameters

**Fig. 1:**
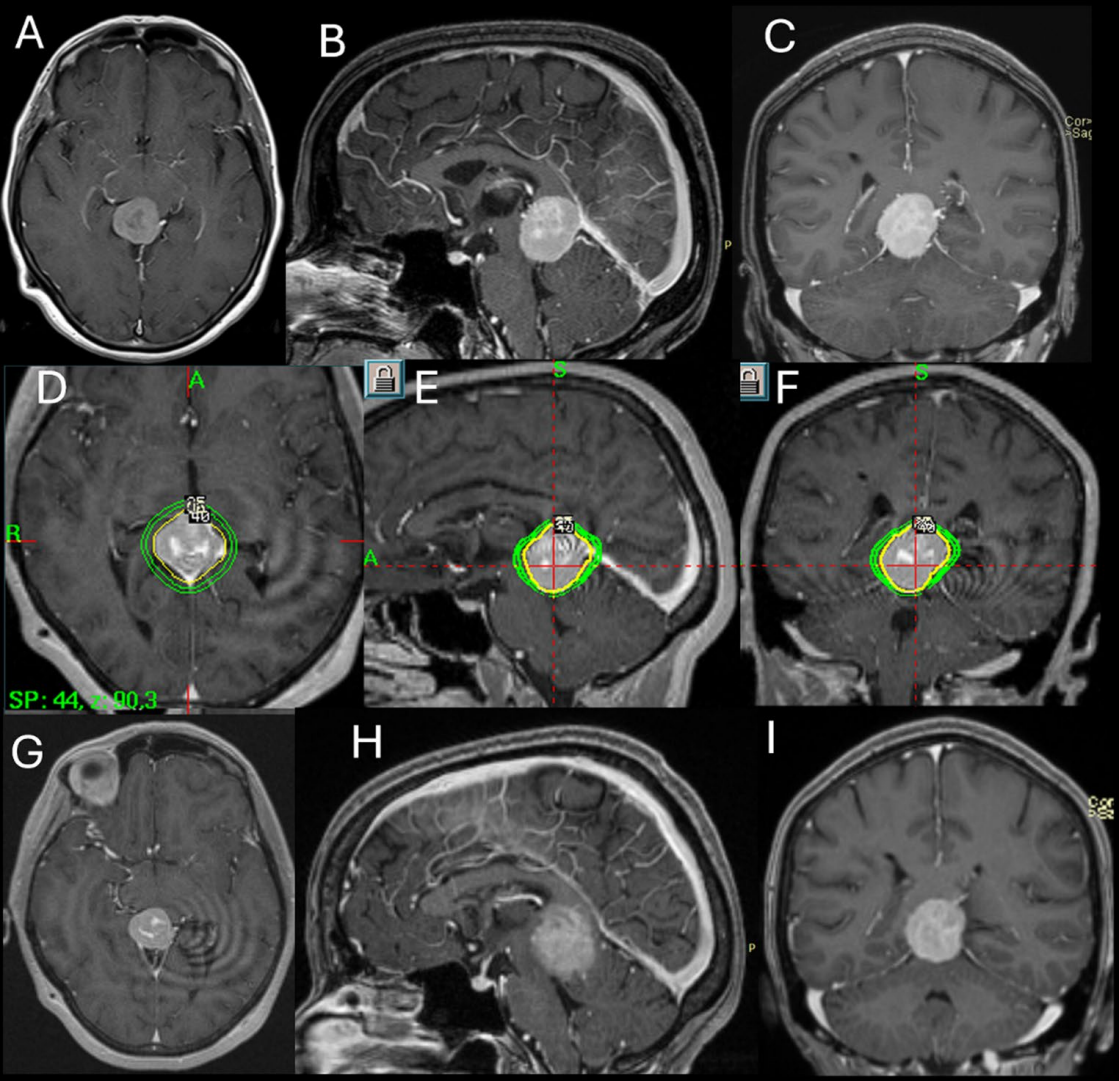
Representative case illustrating the radiosurgical management and long-term radiological outcome of a pineal region meningioma. (**A**, **B**, **C**) Pre-treatment axial, sagittal and coronal contrast-enhanced T1-weighted MRI demonstrating a homogeneously enhancing, 7.8 cm^3^ pineal region meningioma. (**D**, **E**, **F**) Leksell GammaPlan^®^ treatment planning images in axial, sagittal and coronal planes. (**G**, **H**, **I**) Follow-up axial, sagittal and coronal contrast-enhanced T1-weighted MRI obtained 126 months after GKRS demonstrating regressed tumor (6.9 cm^3^). GKRS, Gamma Knife radiosurgery

A total of 44 consecutive patients were included. Baseline characteristics and dosimetric parameters are summarized in Table 1. Female sex predominated (31/44, 70.5%), and mean age was 51.3 ± 11.2 years. Primary GKRS was performed in 35 patients (79.5%), whereas 9 (20.5%) underwent adjuvant GKRS for postoperative residual tumor. Histopathological confirmation was available in 9 of 44 patients (20.5%), all demonstrating WHO Grade I meningioma with Ki-67 ranging from 1% to 5% (reported descriptively). The remaining 35 patients (79.5%) were diagnosed radiologically on the basis of predefined MRI criteria with consensus review by the treating neurosurgeon and neuroradiologist. Sinus or deep venous involvement was present in 40 patients (90.9%), most commonly involving the vein of Galen (26/40, 65.0%) and internal cerebral veins (22/40, 55.0%); 29 of 40 patients (72.5%) had involvement of two or more structures. Baseline peritumoral edema was present in 7 patients (15.9%), and no patient had hydrocephalus at treatment. Median clinical and radiological follow-up was 90 months (IQR 37–106; range 12–174) and 88 months (IQR 37–101; range 12–160), respectively. A representative case is shown in Fig. 1.

**Table 1:** Baseline patient and treatment characteristics

Characteristic	All patients (*n* = 44)	Primary GKRS (*n* = 35)	Adjuvant GKRS (*n* = 9)
**Demographics**			
Age (years), mean ± SD	51.3 ± 11.2	51.5 ± 10.5	50.3 ± 14.1
Age (years), median (IQR)	51 (42–59)	51 (44–59)	47 (36–59)
Sex, female	31 (70.5%)	26 (74.3%)	5 (55.6%)
**Clinical Presentation**			
Presenting symptom			
Headache alone	27 (61.4%)	22 (62.9%)	5 (55.6%)
Headache + vertigo/imbalance	8 (18.2%)	7 (20.0%)	1 (11.1%)
Vertigo/imbalance alone	4 (9.1%)	3 (8.6%)	1 (11.1%)
Other	5 (11.4%)	3 (8.6%)	2 (22.2%)
Symptom duration (months), median (IQR)	18 (6–36)	12 (6–24)	36 (24–60)
Normal neurological examination	40 (90.9%)	33 (94.3%)	7 (77.8%)
KPS at GKRS, median (IQR)	90 (90–100)	100 (90–100)	90 (80–90) p = < 0.001†
**Disease Characteristics**			
GKRS indication			
Primary (first treatment)	35 (79.5%)	35 (100%)	—
Adjuvant (postoperative residual)	9 (20.5%)	—	9 (100%)
WHO Grade I histopathologyᵃ	9/9 (100%)	—	9/9 (100%)
Ki-67 (%), median (IQR)ᵃ	3 (2–4)	—	3 (2–4)
Sinus/deep vein involvement	40 (90.9%)	31 (88.6%)	9 (100%)
Deep vein of Galen	26/40 (65.0%)	20/31 (64.5%)	6/9 (66.7%)
Internal cerebral veins	22/40 (55.0%)	16/31 (51.6%)	6/9 (66.7%)
Straight sinus	18/40 (45.0%)	14/31 (45.2%)	4/9 (44.4%)
Inferior sagittal sinus	14/40 (35.0%)	10/31 (32.3%)	4/9 (44.4%)
Peritumoral edema	7 (15.9%)	1 (2.9%)	6 (66.7%) p = < 0.001†
Hydrocephalus	0 (0%)	0 (0%)	0 (0%)
**GKRS Parameters**			
Tumor volume (cc), median (IQR) [range]	4.75 (2.92–6.88) [0.7–17.7]	4.60 (3.1–5.8)	5.00 (2.5–10.0)
Marginal dose (Gy), median (IQR) [range]	12 (12–13) [11–15]	12 (12–12)	12 (12–14)
Maximum dose (Gy), median (IQR)	26.0 (24.0–30.0)	25.5 (23.3–28.5)	28.0 (26.0–36.0)
Isodose line (%), median (IQR) [range]	50 (45–50) [40–55]	50 (45–50)	50 (45–55)
Number of isocenters, median (IQR) [range]	10 (7–13) [2–32]	10 (7–12)	11 (8–18)
V10 (cc), median (IQR)	7.54 (5.46–12.48)	7.25 (5.28–11.08)	10.15 (5.25–19.73)
V12 (cc), median (IQR)	5.64 (3.68–9.53)	5.63 (3.3–8.4)	9.44 (4.5–16.1)
BED₃ (Gy₃), median (IQR) [range]	60.0 (60.0–69.3) [51.3–90.0]	60.0 (60.0–60.0)	60.0 (60.0–79.3)
**Follow-up**			
Clinical FU (months), median (IQR) [range]	90 (37–106) [12–174]	76 (34–107)	94 (45–108)
Radiological FU (months), median (IQR) [range]	88 (37–101) [12–160]	70 (36–105)	91 (45–100)

BED₃, biologically effective dose (α/β = 3 Gy); FU, follow-up; GKRS, Gamma Knife radiosurgery; IQR, interquartile range; KPS, Karnofsky Performance Scale; V10/V12, brain volume receiving ≥10/≥12 Gy

ᵃ Reported descriptively; available only in the adjuvant (surgically treated) subgroup (n = 9). Ki-67 range: 1–5%

† Statistically significant between-group difference (Mann-Whitney U test for KPS; Fisher’s exact test for peritumoral edema; p < 0.001). Bold red values denote the significantly different subgroup estimates. All other between-group comparisons were non-significant (p > 0.05)

BED₃ is reported for cross-study comparability only; see Statistical Analysis. Sinus and vein percentages calculated among patients with documented involvement (n = 40); individual structures are not mutually exclusive

### Local tumor control

**Fig. 2:**
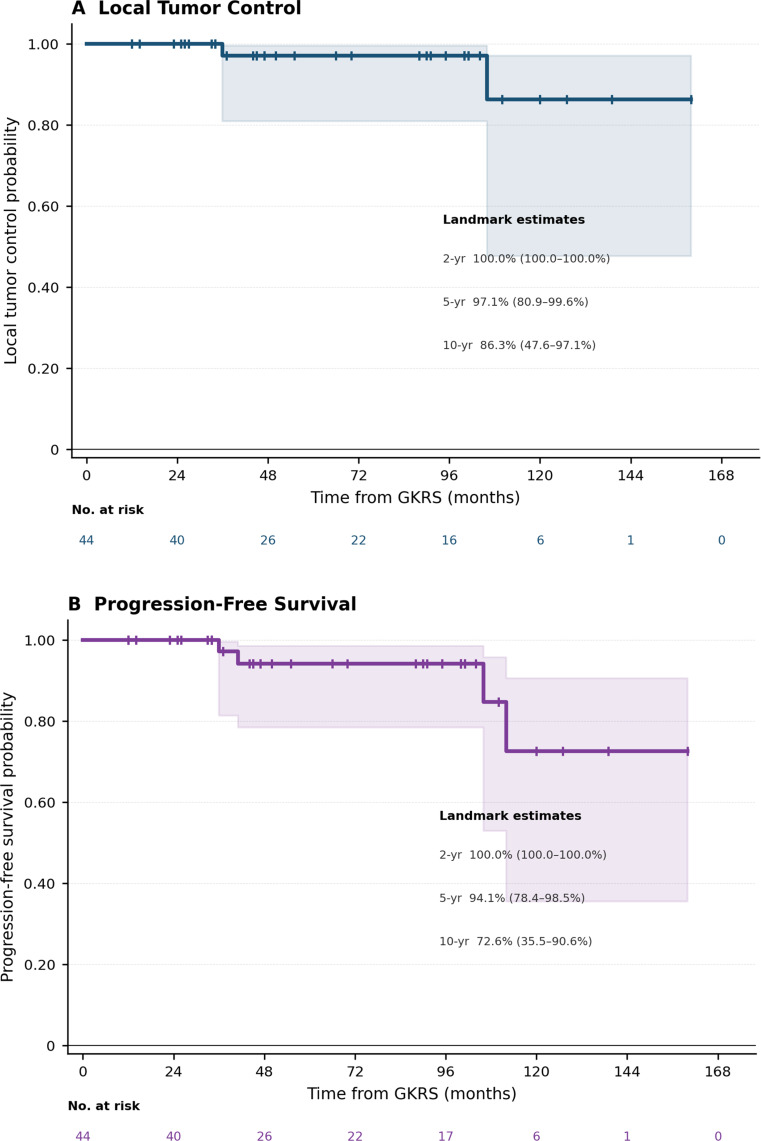

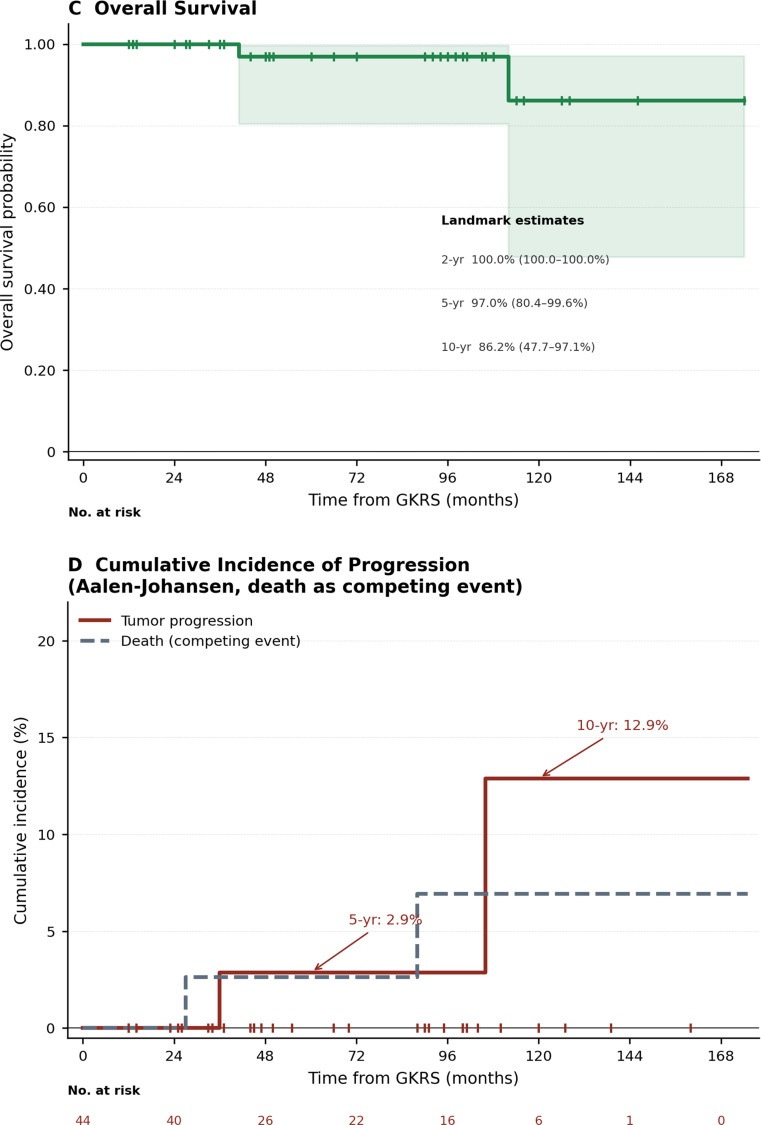
Kaplan-Meier survival estimates and cumulative incidence of tumor progression following single-fraction Gamma Knife radiosurgery for pineal region meningiomas (*n* = 44). (**A**) Local tumor control probability; the event was defined as radiologically confirmed tumor progression, with censoring at last radiological follow-up. (**B**) Progression-free survival; the event was defined as tumor progression or death from any cause (4 events: 2 progressions, 2 deaths), with censoring at last radiological follow-up. (**C**) Overall survival; the event was defined as death from any cause, with censoring at last clinical follow-up. For panels A–C, shaded areas represent 95% confidence intervals (complementary log-log transformation); vertical tick marks indicate censored observations; numbers at risk and actuarial estimates at 2-, 5-, and 10-year landmarks are shown. (**D**) Cumulative incidence function (CIF) for tumor progression estimated by the Aalen-Johansen estimator with death as a competing event (solid red line); death without prior progression is shown as a separate CIF (dashed gray line). Annotated values denote cumulative incidence of progression at 5- and 10-year landmark time points. GKRS, Gamma Knife radiosurgery

**Fig. 3:**
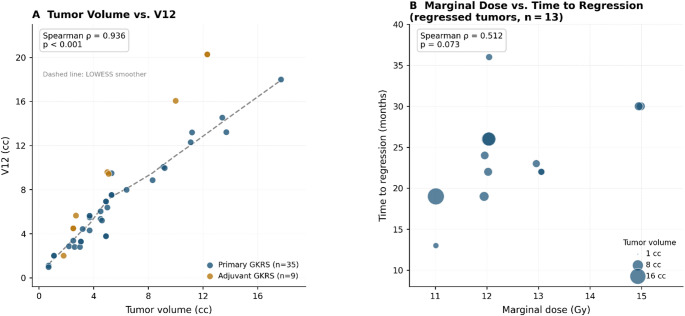
Dose-volume correlations. (**A**) Scatter plot of tumor volume versus V12 (brain volume receiving ≥ 12 Gy) for all 44 patients. Points are color-coded by treatment indication (primary GKRS, blue; adjuvant GKRS for postoperative residual, amber). The dashed line represents a LOWESS smoother shown as a visual guide only. Spearman’s rank correlation coefficient and two-tailed p-value are shown. (**B**) Scatter plot of marginal dose versus time to volumetric regression among the 13 patients who achieved radiological regression. Bubble size is proportional to tumor volume. GKRS, Gamma Knife radiosurgery; V12, brain volume receiving ≥ 12 Gy

At last radiological follow-up, local tumor control was achieved in 42 of 44 patients (95.5%), with regression in 13 (29.5%) and stability in 29 (65.9%) (Table 2). Among patients with regression, median time to volumetric reduction was 23 months (IQR 22–26; range 13–36). Kaplan-Meier local control rates were 100% at 2 years, 97.1% (95% CI 80.9–99.6%) at 5 years, and 86.3% (95% CI 47.6–97.1%) at 10 years (Table 2; Fig. 2A). The Aalen-Johansen cumulative incidence of progression, with death as a competing event, was 0% at 2 years, 2.9% at 5 years, and 12.9% at 10 years (Table 2; Fig. 3).

Two patients (4.5%) developed progression. One patient with a 10.0 cc tumor treated with 14 Gy progressed at 36 months and underwent resection one month after radiological confirmation. The other, with an 8.3 cc tumor treated with 12 Gy, progressed at 106 months and underwent repeat GKRS. Both were event-free at last follow-up. Because only two progression events occurred, progression outcomes are presented descriptively.

**Table 2:** Efficacy and safety outcomes

Outcome	*n* (%) or value
**Tumor Response at Last Radiological Follow-up**	
Regression	13 (29.5%)
Stable	29 (65.9%)
Progression	2 (4.5%)
Local tumor control (regression + stable)	42 (95.5%)
Time to regression (months), median (IQR) [range]	23 (22–26) [13–36]
**Actuarial Local Tumor Control (Kaplan-Meier)**	
2-year	100%
5-year (95% CI)	97.1% (80.9–99.6%)
10-year (95% CI)	86.3% (47.6–97.1%)
**Cumulative Incidence of Progression (Aalen-Johansen**,** death as competing event)**	
2-year	0%
5-year	2.9%
10-year	12.9%
**Actuarial Progression-Free Survival (Kaplan-Meier)**	
2-year	100%
5-year (95% CI)	94.1% (78.4–98.5%)
10-year (95% CI)	72.6% (35.5–90.6%)
**Overall Survival**	
Deaths	2 (4.5%)
Cause unrelated to meningioma/GKRS	2/2 (100%)
2-year OS (95% CI)	100%
5-year OS (95% CI)	97.0% (80.4–99.6%)
10-year OS (95% CI)	86.2% (47.7–97.1%)
**Adverse Radiation Effects**	
Any ARE	3 (6.8%)
Asymptomatic peritumoral edema	1 (2.3%)
Symptomatic peritumoral edema	2 (4.5%)
Onset (months), median [range]	13 [9–14]
Management	
Spontaneous resolution	1
Corticosteroid therapy	2
Permanent ARE-related neurological deficit	0 (0%)
**Functional and Neurological Outcomes**	
KPS at last follow-up, median (IQR)	90 (90–100)
KPS stable	43 (97.7%)
KPS improved	1 (2.3%)
KPS worsened	0 (0%)
Wilcoxon signed-rank test (pre vs. post KPS)	*p* = 0.317
Symptom response at last clinical follow-up	
Improved	18 (40.9%)
Stable	15 (34.1%)
Disappeared	10 (22.7%)
Worsened	1 (2.3%)
New neurological symptoms	0 (0%)
New-onset hydrocephalus	0 (0%)
Distant intracranial lesions	0 (0%)

ARE, adverse radiation effect; CI, 95% confidence interval; GKRS, Gamma Knife radiosurgery; KPS, Karnofsky Performance Scale; OS, overall survival

Kaplan-Meier confidence intervals computed using the complementary log-log transformation

### Overall survival

Two patients died during follow-up (4.5%), both from causes unrelated to their meningioma or GKRS: one from a pancreatic neuroendocrine tumor at 41 months and one from coronary artery disease at 112 months. Kaplan-Meier OS estimates were 100% at 2 years, 97.0% (95% CI 80.4–99.6%) at 5 years, and 86.2% (95% CI 47.7–97.1%) at 10 years (Table 2; Fig. 2C).

### Progression-free survival

PFS, defined as the interval from GKRS to tumor progression or death from any cause, was analyzed among all 44 patients (4 events: 2 progressions, 2 deaths). Kaplan-Meier actuarial PFS rates were 100% at 2 years, 94.1% (95% CI 78.4–98.5%) at 5 years, and 72.6% (95% CI 35.5–90.6%) at 10 years (Fig. 2B).

### Adverse radiation effects

ARE occurred in 3 patients (6.8%): one asymptomatic (peritumoral edema detected on routine MRI at 13 months, managed conservatively) and two symptomatic (onset at 9 and 14 months, both treated with corticosteroids and resolved without sequelae) (Table 2). The first symptomatic patient (tumor volume 5.6 cc, marginal dose 12 Gy) developed edema at 9 months post-GKRS; headache resolved 10 days after initiation of dexamethasone, which was tapered over a 2-month course. The second symptomatic patient (tumor volume 3.3 cc, marginal dose 12 Gy) developed edema at 14 months; headache resolved 15 days after initiation of dexamethasone, tapered over 1 month. Neither patient had residual neurological sequelae at last follow-up. In all three cases, follow-up imaging demonstrated peritumoral edema only, without features suggestive of frank radionecrosis. Alternative etiologies, including tumor progression and venous outflow compromise, were excluded on serial imaging. No patient sustained a permanent ARE-related neurological deficit. V10 and V12 were compared between patients with and without AREs and no statistically significant association was identified. However, this analysis was substantially underpowered given that only three ARE events occurred, and the absence of a significant association should not be interpreted as evidence of no effect.

### Functional and neurological outcomes

KPS was unchanged in 43 patients (97.7%) and improved by 10 points in one (2.3%); no patient experienced deterioration (Wilcoxon signed-rank, *p* = 0.317) (Table 2). Neurological symptoms improved in 18 patients (40.9%), remained stable in 15 (34.1%), and resolved completely in 10 (22.7%); one patient (2.3%) reported symptom worsening, not attributable to ARE. Specifically, no patient developed Parinaud syndrome, new oculomotor nerve palsy (CN III, IV, or VI), tectal or brainstem compression symptoms, new-onset hydrocephalus, or distant intracranial lesions. Formal visual field testing was not routinely performed, and structured gait assessment was limited by the predominant use of teleconsultation for long-term follow-up. All 7 patients with pre-existing peritumoral edema demonstrated resolution on follow-up imaging.

### Subgroup analysis: primary versus adjuvant GKRS

Patients receiving primary GKRS (*n* = 35) and adjuvant GKRS for postoperative residual tumor (*n* = 9) differed significantly in two baseline characteristics: pre-treatment peritumoral edema was present in 6 of 9 adjuvant patients (66.7%) compared with 1 of 35 primary patients (2.9%) (*p* < 0.001), and median KPS at treatment was lower in the adjuvant group (90 [IQR 80–90] vs. 100 [IQR 90–100]; *p* < 0.001), consistent with the functional impact of prior surgical resection. Tumor volume, marginal dose, and radiological follow-up duration did not differ significantly between groups. LC was achieved in 34 of 35 primary patients (97.1%) and 8 of 9 adjuvant patients (88.9%) (Fisher’s exact *p* = 0.371); ARE occurred exclusively in the primary group (3/35, 8.6% vs. 0/9, 0%; *p* = 1.000). Given the small size of the adjuvant subgroup (*n* = 9), the low number of outcome events, and systematic baseline differences between groups, these comparisons are exploratory and lack statistical power to detect clinically meaningful differences. Subgroup analyses by baseline tumor volume, venous involvement pattern, or edema status were not performed, as the event rate precluded any reliable inference.

### Dose-volume correlations

V12 correlated strongly with tumor volume (Spearman ρ = 0.936, *p* < 0.001), reflecting the expected dosimetric relationship (Fig. 3A). A non-significant positive trend was observed between marginal dose and time to regression (ρ = 0.512, *p* = 0.073) (Fig. 3B).

## Discussion

This single-center retrospective study of 44 patients treated with single-fraction GKRS for PMs suggests durable LC, a low rate of AREs, and preservation of functional status over a median radiological follow-up of 88 months. These findings support GKRS as an effective primary and adjuvant treatment option for this anatomically challenging tumor location.

### Comparison with surgical series

Historically, surgical resection has been the primary treatment for PMs. However, achieving GTR is extremely challenging and carries high risks due to the tumor’s close proximity to the deep venous system and the brainstem [5, 8].

Reported surgical resection rates range from 50% to 73%, with substantial morbidity in several series [15]. Moreover, these surgical interventions are associated with significant morbidity and mortality. Raco et al. reported a 23.0% surgical mortality rate and emergency reoperation in 23.0% of surviving patients because of venous infarction or severe edema [8]. Similarly, Zhao et al. [5] reported considerable complication rates, including hemorrhage, hydrocephalus, and neurological deficits, alongside surgical mortality, while Quiñones-Hinojosa et al. [15] documented that all surgically treated patients in their series experienced postoperative visual field defects or transient cortical blindness. These risks have shifted many contemporary surgical strategies toward planned STR followed by adjuvant GKRS for residual tumor [8, 9, 15].

Although no direct surgical comparator was available in this study, the 95.5% LC rate and absence of permanent neurological deterioration compare favorably with published surgical morbidity for this location and suggest a favorable risk-benefit profile for GKRS, particularly in patients with venous involvement.

### Comparison with GKRS literature

Dedicated radiosurgical series for falcotentorial and PMs remain limited [5, 8, 15–17]. Recently, Abdallah et al. reported what was previously the largest series focused exclusively on GKRS for meningiomas of the confluence of the falx and tentorium (CFT) [11]. Their study included 20 patients treated with a median margin dose of 13 Gy for a median tumor volume of 4.4 cc. They achieved exceptional local tumor control rates of 100% at 5 years and 83% at 10 years, with no AREs observed during their surveillance period. Our present study of 44 patients significantly expands on this foundation, representing a more than twofold increase in cohort size and extending follow-up up to 160 months. Our overall LC rate of 95.5%, along with a 5-year PFS of 94.1% and a 10-year PFS of 72.6%, is consistent with their findings and supports the durability of GKRS over an extended period. The 10-year actuarial estimates reported here, including the LC rate of 86.3% and the PFS of 72.6%, are based on only two progression events and four PFS events respectively, and must therefore be interpreted with considerable caution. With fewer than five events beyond the 8-year mark, the Kaplan-Meier curve becomes increasingly unstable and the confidence intervals correspondingly wide; these figures are best understood as hypothesis-generating observations rather than reliable long-term benchmarks.

Similarly, Park et al. reviewed 39 patients with tentorial meningiomas (which included 9 falcotentorial cases) treated with GKRS using a median margin dose of 14 Gy [18]. They reported a 1-year and 5-year PFS of 97% and 92%, respectively, with a 5% rate of symptomatic AREs. Our cohort’s 6.8% ARE rate and comparable long-term PFS directly align with these results, consistent with the dose ranges reported in comparable series, though firm conclusions regarding dose selection cannot be drawn from the present data.

In the broader context of pineal region tumors, a recent systematic review and pooled analysis by Gagliardi et al. evaluated primary GKRS across various histological subtypes [10]. They found that benign, low-grade lesions in this region respond exceptionally well to radiosurgery, suggesting that primary GKRS may offer effective LC with an acceptable toxicity profile in carefully selected patients.

Taken together, these comparisons support the role of GKRS as both primary treatment and adjuvant therapy after STR [5, 15, 19]. Studies by Zhao et al. and Talacchi et al. have demonstrated that leaving residual tumor adherent to the deep veins and subsequently treating it with radiosurgery minimizes permanent neurological deficits while ensuring long-term tumor stability [5, 19]. Our data is consistent with this emerging multidisciplinary approach. While favorable outcomes were observed in both primary and adjuvant subgroups, the small adjuvant sample and low event rates preclude any conclusion regarding the equivalence of these indications. Prospective comparative studies with adequate sample sizes are needed to define the relative efficacy of primary versus adjuvant GKRS for PMs.

### Adverse radiation effects

ARE occurred in 3 patients (6.8%), all of whom recovered without permanent sequelae. No patient developed new persistent neurological deficits, prolonged steroid dependence, or imaging evidence of aqueductal or ventricular compromise.

In the previously largest series of CFT meningiomas, Abdallah et al. reported an exceptional safety profile, observing absolutely no AREs among 20 patients treated with a median margin dose of 13 Gy over a median MRI surveillance of 59 months [11]. Similarly, Park et al. evaluated 39 patients with tentorial meningiomas treated with a slightly higher median marginal dose of 14 Gy [18]. They observed asymptomatic peritumoral edema in 5% of patients and symptomatic AREs in another 5%.

In a broader systematic review and pooled analysis of primary GKRS for pineal region tumors, Gagliardi et al. reported a radionecrosis rate of 6% and an overall treatment-related complication rate of 10.5% [10]. Consistent with our experience, the vast majority of these complications were effectively managed with medical therapy, particularly corticosteroids. When examining adjacent deep-seated lesions, such as intraventricular meningiomas, Samanci et al. noted a single case of symptomatic peritumoral edema among 6 patients treated with a median dose of 12 Gy, which completely resolved following a 4-week steroid treatment [20].

Broadly, general ARE rates for intracranial meningiomas treated with GKRS typically range from 4.6% to 23.6% across different studies [11]. The marginal doses used here, median 12 Gy, range 11 to 15 Gy, also fall within the range reported for comparable deep-seated meningioma series, although the present dataset does not permit dose optimization analysis.

### Functional outcomes

In our cohort, functional outcomes following single-fraction GKRS were notably favorable. No patient developed a new, permanent neurological deficit, and functional status remained stable or improved in 100% of cases.

These results contrast starkly with the functional decline frequently observed after microsurgical resection. Surgical corridors, such as the occipital transtentorial approach, demand prolonged brain retraction that routinely puts visual pathways at risk [5]. Consequently, transient or permanent visual field defects and cortical blindness are highly common surgical complications, with some series reporting visual impairments in all patients immediately postoperatively [15]. Furthermore, surgical dissection around the delicate deep venous system carries a profound risk of causing severe cognitive and motor deficits. Intraoperative injury to the deep veins or their collateral channels can lead to devastating consequences, including permanent memory disturbance, hemiparesis, and dysarthria, which drastically diminish the patient’s quality of life [5, 21].

### Limitations

This study has several limitations. First, its retrospective single-center design introduces selection bias, and patients treated with GKRS likely represent a more favorable subgroup than the broader surgical population. Second, histopathological confirmation was available in only 9 of 44 patients (20.5%), while the remaining 35 (79.5%) were diagnosed radiologically. Although all non-operated lesions met predefined imaging criteria and underwent consensus neuroradiological review, misclassification with potential mimics cannot be fully excluded. These entities may exhibit different volumetric behavior and LC rates following radiosurgery. Furthermore, combining these radiologically diagnosed cases with histologically confirmed postoperative residual tumors introduces biological heterogeneity, and the small adjuvant subgroup precluded meaningful between-group inference. Nevertheless, the high rate of long-term tumor stability observed in this series is consistent with the expected behavior of WHO Grade I meningiomas and provides indirect support for the radiological diagnoses. Third, the low number of outcome events precluded multivariable modeling and formal identification of predictors. Readers should therefore not infer causal or predictive relationships from the exploratory dose–volume correlations presented, since these associations are hypothesis-generating only. Fourth, follow-up imaging was acquired on both 1.5T and 3T scanners without standardized protocols, and formal intra- and interobserver volumetric reliability was not assessed. The ± 20% response thresholds, while consistent with prior literature [13, 14], are susceptible to measurement variability from differences in field strength, slice thickness, and segmentation technique. Tumors near the classification boundary may be misassigned between “stable” and “regression” categories, and time-to-regression estimates may partly reflect detection latency. Fifth, follow-up duration was heterogeneous, which may have further influenced response classification and survival estimates. Finally, the absence of an institutional surgical comparator precludes direct treatment-effect comparison within the same cohort.

## Conclusion

Single-fraction GKRS achieves durable LC with a low rate of AREs and preservation of neurological function in patients with PMs. These results support GKRS as a safe and effective primary treatment strategy for this surgically challenging tumor location. Preliminary observations in the adjuvant subgroup are encouraging but require validation in larger cohorts. Prospective multicenter studies with standardized volumetric follow-up protocols are needed to establish definitive long-term benchmarks and identify predictors of outcome in this rare tumor subgroup.

## Data Availability

No datasets were generated or analysed during the current study.

## References

[1] Konovalov AN, Spallone A, Pitzkhelauri DI (1996) Meningioma of the pineal region: a surgical series of 10 cases. J Neurosurg 85:586–590. 10.3171/jns.1996.85.4.0586 8814160 10.3171/jns.1996.85.4.0586

[2] de Blasco García G, Delgado-Fernández J, Penanes Cuesta JR et al (2019) Meningiomas Originated at the Falcotentorial Region: Analysis of Topographic and Diagnostic Features Guiding an Optimal Surgical Planning. World Neurosurg 123:e723–e733. 10.1016/j.wneu.2018.12.013 30580064 10.1016/j.wneu.2018.12.013

[3] Yu L, Orazmyradov B, Qi S et al (2020) Reinvestigation of the origins of pineal meningiomas based on its related veins and arachnoid membranes. BMC Neurol 20:200. 10.1186/s12883-020-01783-4 32434477 10.1186/s12883-020-01783-4 PMC7238570

[4] Nowak A, Dziedzic T, Czernicki T et al (2014) Falcotentorial and velum interpositum meningiomas: two distinct entities of the pineal region. Neurol Neurochir Pol 48:397–402. 10.1016/j.pjnns.2014.09.009 25482250 10.1016/j.pjnns.2014.09.009

[5] Zhao X, Belykh E, Przybylowski CJ et al (2020) Surgical treatment of falcotentorial meningiomas: a retrospective review of a single-institution experience. J Neurosurg 133:630–641. 10.3171/2019.4.JNS19208 31374550 10.3171/2019.4.JNS19208

[6] Dalle Ore CL, Magill ST, McDermott MW (2020) Falcotentorial meningiomas. Handb Clin Neurol 170:107–114. 10.1016/B978-0-12-822198-3.00033-1 32586482 10.1016/B978-0-12-822198-3.00033-1

[7] Chandy MJ, Damaraju SC (1998) Benign tumours of the pineal region: a prospective study from 1983 to 1997. Br J Neurosurg 12:228–233. 10.1080/02688699845041 11013685 10.1080/02688699845041

[8] Raco A, Agrillo A, Ruggeri A et al (2004) Surgical options in the management of falcotentorial meningiomas: report of 13 cases. Surg Neurol 61:157–164 discussion 164. 10.1016/s0090-3019(03)00573-1 14751629 10.1016/s0090-3019(03)00573-1

[9] He W, Chen Z, Xu C et al (2024) Twelve-year experience of pineal region meningiomas: long-term outcomes of maximal safe resection. Neurosurg Rev 47:822. 10.1007/s10143-024-03069-6 39453537 10.1007/s10143-024-03069-6

[10] Gagliardi F, De Domenico P, Garbin E et al (2023) Primary Gamma Knife Radiosurgery for pineal region tumors: A systematic review and pooled analysis of available literature with histological stratification. J Pineal Res 75:e12910. 10.1111/jpi.12910 37705383 10.1111/jpi.12910

[11] Abdallah HM, Mallela AN, Wei Z et al (2023) Gamma Knife radiosurgery for meningiomas of the confluence of the falx and tentorium. J Neurooncol 161:225–233. 10.1007/s11060-022-04125-1 36125641 10.1007/s11060-022-04125-1

[12] Benedict SH, Yenice KM, Followill D et al (2010) Stereotactic body radiation therapy: the report of AAPM Task Group 101. Med Phys 37:4078–4101. 10.1118/1.3438081 20879569 10.1118/1.3438081

[13] Chukwueke UN, Wen PY (2019) Use of the Response Assessment in Neuro-Oncology (RANO) criteria in clinical trials and clinical practice. CNS Oncol 8:CNS28. 10.2217/cns-2018-0007 30806082 10.2217/cns-2018-0007 PMC6499019

[14] Yang D-Y, Sheehan J, Liu Y-S et al (2009) Analysis of factors associated with volumetric data errors in gamma knife radiosurgery. Stereotact Funct Neurosurg 87:1–7. 10.1159/000177622 19039257 10.1159/000177622

[15] Quiñones-Hinojosa A, Chang EF, Chaichana KL, McDermott MW (2009) Surgical considerations in the management of falcotentorial meningiomas: advantages of the bilateral occipital transtentorial/transfalcine craniotomy for large tumors. Neurosurgery 64:260–268 discussion 268. 10.1227/01.NEU.0000344642.98597.A7 19287325 10.1227/01.NEU.0000344642.98597.A7

[16] Okami N, Kawamata T, Hori T, Takakura K (2001) Surgical treatment of falcotentorial meningioma. J Clin Neurosci 8 Suppl 1:15–18. 10.1054/jocn.2001.0870 10.1054/jocn.2001.0870 11386819

[17] Hong CK, Hong JB, Park H et al (2016) Surgical Treatment for Falcotentorial Meningiomas. Yonsei Med J 57:1022–1028. 10.3349/ymj.2016.57.4.1022 27189300 10.3349/ymj.2016.57.4.1022 PMC4951445

[18] Park S-H, Kano H, Niranjan A et al (2015) Gamma Knife radiosurgery for meningiomas arising from the tentorium: a 22-year experience. J Neurooncol 121:129–134. 10.1007/s11060-014-1605-0 25186087 10.1007/s11060-014-1605-0

[19] Talacchi A, Biroli A, Hasanbelliu A, Locatelli F (2018) Surgical Management of Medial Tentorial Meningioma: Falcotentorial and Torcular. World Neurosurg 115:e437–e447. 10.1016/j.wneu.2018.04.066 29678716 10.1016/j.wneu.2018.04.066

[20] Samanci Y, Oktug D, Yilmaz M et al (2020) Efficacy of gamma knife radiosurgery in the treatment of intraventricular meningiomas. J Clin Neurosci 80:38–42. 10.1016/j.jocn.2020.08.016 33099364 10.1016/j.jocn.2020.08.016

[21] Goto T, Ohata K, Morino M et al (2006) Falcotentorial meningioma: surgical outcome in 14 patients. J Neurosurg 104:47–53. 10.3171/jns.2006.104.1.47 16509146 10.3171/jns.2006.104.1.47

